# Sampling methods for flexible endoscopes without a working channel: a scoping review

**DOI:** 10.1017/ice.2025.56

**Published:** 2025-06

**Authors:** Yana Halmans, David Wellenstein, Joost Hopman, Robert Takes, Guido van den Broek

**Affiliations:** 1 Department of Otorhinolaryngology and Head and Neck Surgery, Radboud University Medical Center Nijmegen, P/O Box 9101, Nijmegen, The Netherlands; 2 Institute for Patientcare, Radboud University Medical Center Nijmegen, P/O Box 9101, Nijmegen, The Netherlands

## Abstract

**Background:**

A diagnostic flexible laryngoscopy using a flexible endoscope (FE) without a working channel can become contaminated when inserted through the nose to inspect the throat. Microbiological surveillance is necessary to ensure adequate reprocessing. A lack of knowledge exists about the most accurate way to assess microbiological contamination on the surface of FEs without a working channel. A scoping review of research on sampling techniques for FEs without a working channel was done to identify frequently used sampling techniques and to determine the best way to assess microbiological contamination.

**Methods:**

PubMed, Embase, Cochrane Library, and CINAHL databases were searched. Data related to the sampling technique and bacterial contamination were extracted.

**Results:**

Twelve of the 378 studies met the inclusion criteria. None compared sampling techniques, most studies investigated the efficacy of several disinfection methods. Retrieved sampling techniques were immersion, swabbing, and wiping. Immersion and wiping could detect bacterial contamination on contaminated FEs without a working channel. Two out of six studies using a swabbing method found bacterial contamination on contaminated FEs without a working channel. Three studies using the swabbing method detected bacterial contamination after disinfection. One study did not retrieve microorganisms after disinfection using the swabbing method.

**Conclusions:**

Three different sampling techniques were extracted: immersion, wiping, and swabbing, which could all detect microbiological contamination on contaminated FEs without a working channel. However, this scoping review identified significant gaps in literature. Additional research is needed to determine the best sampling technique(s) for FEs without a working channel to detect microbiological contamination.

## Introduction

A diagnostic flexible laryngoscopy (ie flexible endoscopy) is performed with a flexible endoscope (FE) (ie flexible laryngoscope) without a working channel. A diagnostic flexible laryngoscopy is one of the most performed procedures within otorhinolaryngology (ORL) and is crucial in the diagnostic process.^1^ The number of diagnostic flexible laryngoscopies has increased by 87% from 2000 to 2016.^2^ In 2016, otorhinolaryngologists performed 575355 diagnostic flexible laryngoscopies in the United States Medicare population.^2^ It can be assumed that this number has further increased since previous research and organizations, such as the American Academy of Otolaryngology-Head and Neck Surgery (AAO-HNSF), stressed the importance of performing a diagnostic flexible laryngoscopy in addition to taking a medical history and performing a physical examination.^3,4^ For example, a diagnostic flexible laryngoscopy is used to visualize the pharynx or larynx for diagnosing disorders involving difficulties with swallowing, vocal issues or diseases of the upper aerodigestive tract.^2^ Previous research showed that FEs can become contaminated with microorganisms, blood, and secretions during clinical use.^5–7^ As the FE without a working channel is used in multiple patients daily, it carries the risk of cross-contamination between patients, risking outbreaks of healthcare-related infections (HAI). Transmission of pathogens has been described following colonoscopy, gastrointestinal endoscopy, and flexible bronchoscopy.^8^ Healthcare-associated infections in flexible endoscopy are low at one case per 1.8 million procedures.^9^

Professional organizations have developed several guidelines for reprocessing FEs without a working channel to prevent transmission of pathogens.^10–14^ Routine microbiological surveillance is necessary to ensure that the reprocessing of FEs is performed adequately. However, the guidelines for reprocessing endoscopes mainly focus on FEs with a working channel and provide limited or conflicting information about assessing microbiological contamination on FEs without a working channel. A frequently suggested method is collecting liquid samples from the working channels, which is only possible for FEs with a working channel. The NEN-EN-ISO 15883-5, which specifies procedures and test methods used to demonstrate the cleaning efficacy of washer-disinfectors (WD) and their accessories, suggests periodic determination of the effectiveness of the machine cleaning process using test soiling.^15^ The European Society of Gastrointestinal Endoscopy and European Society of Gastroenterology and Endoscopy Nurses and Associates (ESGE-ESGENA) guideline recommends a swab of the outer surface of the endoscopes to assess microbiological contamination.^12^ Other guidelines provide no information about assessing microbiological contamination on a FE. Other methods described in literature include sampling via contact plates, dipping the FE into a sterile culture medium, and wiping moistened swabs, sponges, or sterile gauzes over the surface of the FE.^16–19^ Although protocols for sampling channeled endoscopes exist, there is no consensus for FEs without a working channel.

The following research questions were formulated:^1^ What existing sampling techniques are known from the literature for sampling the shaft and tip of contaminated flexible endoscopes without a working channel?^2^ Which sampling techniques can determine microbiological contamination (ie, Colony Forming Units (CFUs)) on the shaft and tip of contaminated flexible endoscopes without a working channel?

## Methods

### Protocol and registration

The review process followed the framework suggested by Arksey and O’Malley and the revisions by Levac et al..^20,21^ This study was reported using the Preferred Reporting Items for Systematic reviews and Meta-Analyses extension for Scoping Reviews (PRISMA-Scr) (supporting information 1).^22^

### Eligibility criteria

Studies included in this review needed to focus or report information on sampling techniques for FEs without a working channel. There was no limitation concerning the date of publication. Papers had to be written in Dutch or English. Papers were excluded when the focus was not on FEs without a working channel, for example, when papers focused on endoscopes with a working channel or when the full text was unavailable.

### Information sources and search

PubMed, Embase, Cochrane Library, and CINAHL databases were systematically searched to identify potentially relevant documents. The search strategies were drafted by an experienced librarian and further refined through team discussion. The final search strategy for the selected databases can be found in supporting information 2. The last search was performed in October 2022. The final search results were exported into EndNote, where duplicates were removed. Reference lists of included papers were reviewed to identify further relevant studies.

### Selection of sources of evidence/study selection

**Figure 1.**
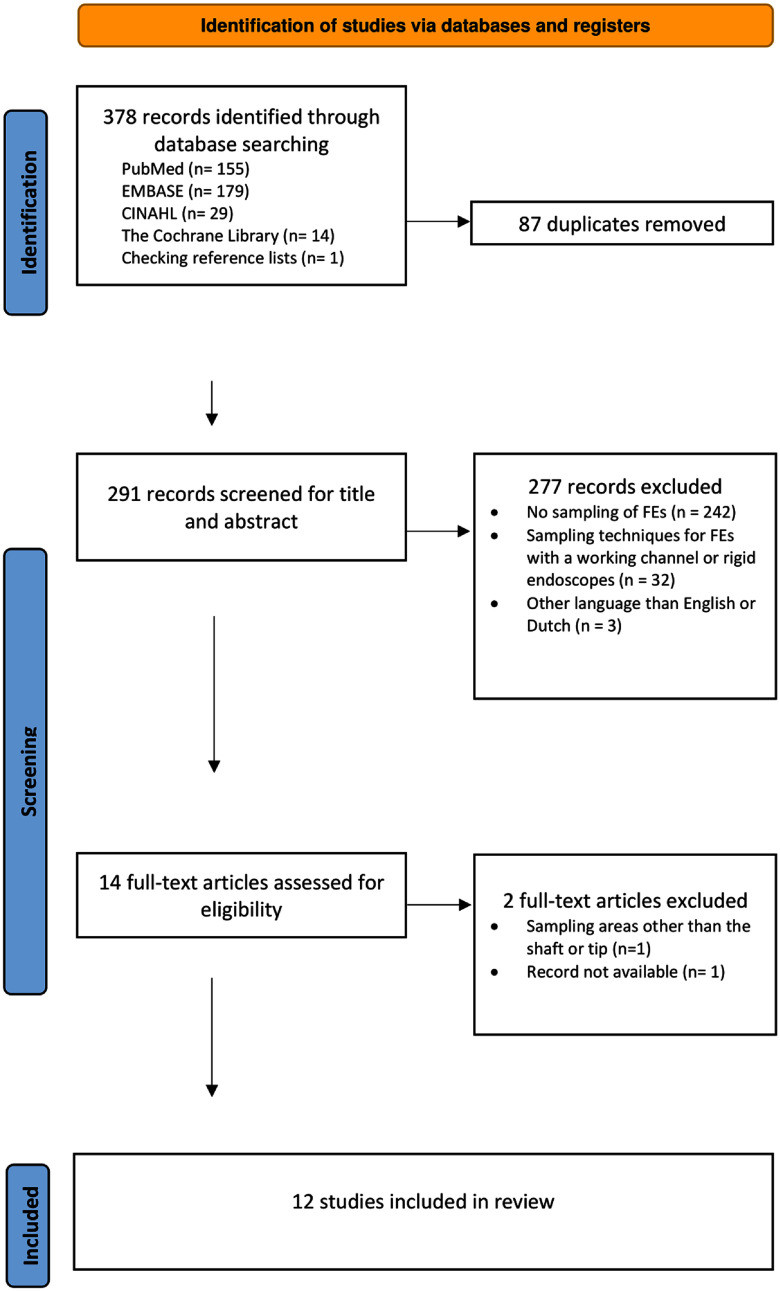
A Preferred Reporting Items for Systematic reviews and Meta-Analyses flow diagram of the study selection process.

After excluding duplicates, two authors (YH, DW) independently reviewed the retrieved citations for possible eligibility by performing a title and abstract screening based on the selection criteria (table 1). The full text was extracted for full-text screening for citations identified for possible inclusion. Discrepancies were resolved by discussion or consulting a senior author (GB). The PRISMA search flow summarizes the study selection strategy (figure 1).

**Table 1.** Inclusion/exclusion criteria

Inclusion criteria	
Type of study	Randomized/non-randomized trials, cohort studies, cross-sectional, case control, prospective, retrospective, case series, case reports, reviews, in vitro
Cohort	Flexible endoscopes without a working channel
Language	English or Dutch
**Exclusion criteria**	
Type of study	Letters, technical notes, conference documents, book chapters, surveys
Cohort	Flexible endoscopes with a working channel, rigid endoscopes, laryngoscopes used for oral intubation
Language	**Other than English or Dutch**

### Data charting process and data items

One reviewer (YH) extracted the data using a data-charting form created by two reviewers (YH, DW). The following variables were extracted and listed: authors, year of publication, country, study design, objective(s), endoscope type, sampling technique(s), moment of sample taking, key findings or opinions related to sampling techniques of a FE without a working channel, bacteria found, culture plate and incubation time.

## Results

### Literature search

Three hundred seventy-eight articles were identified from databases and reference lists, of which 291 were original studies (figure 1). These were screened based on title, abstract, and full text. A total of 12 studies published between 1993 and 2022 were included.

### Study description

The primary study characteristics are summarized in Table 2. Details on individual studies regarding the sampling technique, moment of sample taking, culture plate, incubation time, identified bacteria, and key findings related to each sampling technique are presented in supporting information 3. The number of studies available per sampling technique varied from two to six.

**Table 2.** Study characteristics number of studies

Date	
1993 – 2000	1
2001 – 2010	3
2011 – 2022	8
**Study design**	
Controlled clinical trial	5
Cross-sectional	5
In vitro	2
**Country**	
Italy	2
Japan	1
New Zealand	1
United Kingdom	2
United States	6
**Sampling technique** ^1^	
Immersion/dipping	5
Swabbing	6
Wiping	2
**Sample size (studies combined)**	
Immersion/dipping	373
Swabbing	1224
Wiping	162

1 More than total studies because Ditommaso et al. used two different sampling techniques.

The studies were conducted in five different countries. Six studies originated from The United States.^19,23–27^ Two studies were conducted in Italy^28,29^ and two in the United Kingdom.^18,30^ One study was conducted in New Zealand^31^ and one in Japan.^32^ None of the studies compared sampling techniques for FEs without a working channel as a primary objective. Most studies (n=10) investigated the efficacy of several disinfection methods for FEs without a working channel. The two remaining studies investigated the efficacy and reliability of a sheath, which can be easily applied over a FE before performing the endoscopic procedure to prevent cross-contamination.^24,26^

### Sampling techniques

Most studies used only one sampling technique (n=11), and one study used two sampling techniques since a different technique was used for the tip than for the shaft.^29^ Most studies did not differentiate between sampling of the tip and the shaft of the FE.^19,25,27,28^ Two studies only sampled the tip.^23,30^ Two studies only sampled the head and shaft.^24,26^ Two studie sampled the tip and handle.^18,31^ Okano et al. differentiated between the tip, shaft and handle.^32^ Three sampling techniques were identified: immersion (ie, dipping), wiping, and swabbing. The most commonly described sampling technique was swabbing with a sterile swab (n = 6), followed by immersing or dipping the tip of the FE without a working channel in a culture medium (n = 5) and wiping the surface using a moistened wipe (n = 2). The exact culture medium used when immersing or dipping the FE without a working channel in a culture medium mainly was not specified and described as a neutralizing buffer (n = 1), a sterile culture medium (n = 1), sterile saline (n = 1), a preservation medium (n = 1) and tryptic soy broth (n = 1).

### Moment of sampling

The moment of sampling varied per study. Mostly, multiple samples were taken at different times. In most studies, samples were taken after clinical examination or laboratory contamination and after disinfection (n = 6). Other moments of sampling were prior to application of the sheath and clinical examination, after examination and sheath removal, and after disinfection (n = 1), prior to clinical examination, after control contamination and after disinfection (n = 1), after disinfection and after storage for <72 or >72 hours (n = 1), after disinfection (n = 1), before in vitro contamination and after disinfection (n = 1) and directly after disinfection and between the cleaning process and application (n = 1).

### Culture plates and incubation times

Most frequently, the culture plate used was not specified (n = 5). In four studies, multiple types of agar plates and selective agar plates were used (see supporting information 3 for a specification for the used selective agar plates). Abramson et al. used blood agar, chocolate agar, Mitis-Salivarius agar, and Selective Enterococcus for culturing.^23^ Alvadaro et al. used 5% sheep blood agar.^24^ Elackattu et al. used blood agar plates.^26^ Liming et al. used chocolate agar or Sabouraud dextrose plates.^27^ Cottarelli et al. used selective agar plates (supporting information 3). Six studies did not specify the culture plates used.^18,19,29–32^ The incubation time of the culture plates was not specified in 5 studies or varied between 24-48 hours (n = 3), 48 hours (n = 2), and 72 hours (n = 2). The incubation temperature mainly was not specified (n = 7), followed by 35°C (n = 2), 37°C (n = 2), and 36°C (n = 1).

### Key findings related to the detection of contamination on FEs

All studies using the immersion sampling technique were able to detect microorganisms after clinical examination or laboratory contamination and after several ways of (high-level) disinfection.^19,23,25,27,29^ Two studies using the wiping sampling technique could detect microorganisms after clinical contamination, but only the study of Ditommaso et al. detected microorganisms after high-level disinfection (HLD)^24,29^ The swabbing sampling technique could detect microorganisms after HLD in three studies.^28,30,31^ The study by Elackattu et al. showed no microorganisms after disinfection with 70% alcohol-soaked gauze.^26^ In the study of Tzasinksi et al., the swapping sampling technique was only able to yield microorganisms after HLD at the handle of the endoscope; no microorganisms were detected at the tip.^18^ Two studies provided information about bacterial detection using the swabbing sampling technique after clinical contamination.^26,32^

### Detected bacteria

Only the study performed by Liming et al. did not specify the bacteria found after incubation.^27^ The microorganisms detected were Gram-positive bacilli, Gram-positive cocci, Gram-negative bacilli. A more detailed overview of the detected microorganisms can be found in supporting information 3.

## Discussion

### Summary of evidence

To our knowledge, this is the first scoping review to comprehensively review the studies on sampling FEs without a working channel. The current review identified three different sampling techniques described in the literature. Our findings indicate that limited to no research is available in this area, and several sampling techniques are used to detect microorganisms on FEs without a working channel. Inconsistencies in study design and often incomplete reporting of culture processing methods make it difficult to compare across studies. However, the studies identified incorporate sampling techniques and bacterial contamination for FEs.

The first objective of this study was to seek an answer to the following question: What are the existing sampling techniques for sampling the shaft and tip of contaminated FEs without a working channel? The results indicate that three different sampling techniques are used to determine contamination on FEs without a working channel. Six studies used a swab to assess microbiological contamination, four used an immersion or dipping method, and one investigated bacterial contamination using a moistened wipe. One study used both a moistened wipe as an immersion method.

The study’s second objective was to determine which sampling techniques could determine microbiological contamination (ie, Colony Forming Units (CFUs)) on the shaft and tip of contaminated FEs without a working channel. Only Okano et al. evaluated the contamination of both the shaft and the tip and found no difference in contamination after disinfection.^32^ The immersion technique was able to determine bacterial contamination on clinically or in vitro contaminated FEs without a working channel in all studies using the immersion technique. The immersion technique also detected microbiological contamination on FEs without a working channel after several ways of (high-level) disinfection.^19,23,25,27,29^ Both studies using the wiping sampling technique showed that this method could detect microbiological contamination on contaminated FEs without a working channel.^24,29^ Ditommaso et al. found that the wiping sampling technique could also yield microorganisms after HLD.^29^ Only two of the six studies using the swabbing sampling technique checked for bacterial contamination after clinical contamination. Both studies detected microbiological contamination on FEs without a working channel after clinical contamination.^26,32^ The remaining four studies using the swabbing sampling technique provided no information about bacterial contamination after clinical or in vitro contamination. However, the swabbing sampling technique detected microorganisms after HLD or disinfection with 70% alcohol-soaked gauze.^26,28,30,31^ This suggests that the swabbing sampling technique would also be able to detect microorganisms on a contaminated FE without a working channel when, presumably, the bacterial contamination will be higher than after disinfection. Only in the study of Tzasinksi et al. was the swabbing sampling technique not able to yield microorganisms at the tip or shaft of the FE without a working channel.^18^

Two studies investigated contamination of FEs after using a sheath. Since this serves as an extra cover while performing the laryngoscopy, it is likely to assume that the contamination is less than it would be without the use of a sheath.^24,26^

To evaluate the adherence of microorganisms to different materials, Abramson et al. contaminated a FE, rigid endoscope, and glass rod in vitro with S. aureus, E. coli, pseudomonas aeruginosa, S. sanguis, and C. albicans.^23^ Although the adherence varied per material and microorganism, the immersion sampling technique was sensitive enough to retrieve microorganisms of all three different materials. In the study of Chang et al., a FE was in vitro contaminated with S. aureus, previously cultured in a liquid medium, or C. albicans, previously cultured in Sabouraud dextrose broth, to serve as a positive control.^25^ Chang et al. showed that the positive controls stayed positive, indicating that the sensitivity of the sampling technique used in this study, immersion, was good enough to detect microbiological contamination. In the study of Phua et al., the FEs were in vitro contaminated with S. epidermidis.^30^ Since most studies were clinical studies, the degree of contamination after clinical contamination was unknown. Therefore, it is difficult to assess the sensitivity of the sampling techniques.

None of the identified studies elaborated on the practical implementation of the sampling techniques. In our opinion, this is also an important factor to include when choosing the preferred sampling technique for FEs without a working channel. Practical difficulties, such as required personnel, specific areas allocated to sample taking, or necessary conditions under which the culture is taken, are factors to consider when implementing a sampling technique. Although the immersion technique was able to yield microorganisms after HLD, it may be more difficult to perform an adequate immersion technique versus a swabbing technique since there are more steps involved in the immersion technique.

In addition, costs associated with the sampling techniques were not mentioned. However, this might also be another factor in deciding the best sampling techniques for FEs. Costs may differ per sampling technique since different materials are being used, but they may also depend on the staff necessary for the sample taking. Examples of costs associated with sampling are the culture medium needed, the materials used for sampling the material (eg, swabs, wipes), incubation devices, personnel needed for the sampling taking or for the evaluation of the cultures, transportation costs, personnel protection materials (eg, gloves, masks) etcetera.

### Limitations

The review was limited by a small number of studies and an even smaller number of studies using a randomized controlled design because of limited research in this area. In addition, the identified studies often consisted of a small sample size. None of the studies had identifying the best sampling technique for FE without a working channel as its objective, resulting in a lack of control group, information and clarity for some steps of the sample-taking process. The methods used for the sampling and the incubation process were mostly poorly described. In addition, most studies did not describe the time between clinical use and the sample taking. A great variety of FEs were used in the different studies, and the type of FE used was rarely well described, making it difficult to assess whether this influences the determination of the best sampling technique.

## Conclusions

The lack of evidence to support one of the sampling techniques for FEs without a working channel poses a challenge when wanting to sample FEs without a working channel for assessing microbiological contamination. Although limited, evidence was found for both the immersion, wiping, and swabbing techniques that they could detect bacteria on FEs without a working channel. Therefore, additional research is needed to identify the best sampling technique for FEs without a working channel to determine microbiological contamination.

## Supporting information

Halmans et al. supplementary material 1 Halmans et al. supplementary material

Halmans et al. supplementary material 2 Halmans et al. supplementary material

Halmans et al. supplementary material 3 Halmans et al. supplementary material

## Supplementary material

The supplementary material for this article can be found at https://doi.org/10.1017/ice.2025.56
