# Supplementary material for: Sampling methods for flexible endoscopes without a working channel: a scoping review
Source: Infect Control Hosp Epidemiol. 2025 Apr 21;46(6):635–40. doi: 10.1017/ice.2025.56 (PMC12169950; doi:10.1017/ice.2025.56)
Supplement: Halmans et al. supplementary material 2 — Halmans et al. supplementary material [file S0899823X2500056Xsup002.docx]

***Supporting information 2: Search strategy***

**PubMed: 155**

(("Laryngoscopes"[Mesh] OR "Laryngoscopy"[Mesh] OR (("Endoscopes "[Mesh] OR Endoscop*[tiab]) AND (Ear-Nose-Throat[tw] OR ENT[tw] OR Otolaryngo*[tw] OR Otorhinolaryngo*[tw] OR pharynx[tw] OR larynx[tw] OR nasal[tw] OR Laryngoscop*[tiab] OR Pharyngolaryngoscop*[tiab] OR Pharyngoscop*[tiab] OR Otorhinolaryngoscop*[tiab]))) AND ("Culture Techniques"[Mesh] OR "microbiology"[Subheading] OR Culture[tiab] OR Cultures[tiab] OR Culturing[tiab] OR Cultivation[tiab] OR Sample-tak*[tiab] OR Sample-collect*[tiab] OR samples[tiab] OR sampling-method*[tiab] OR Wipes[tiab] OR Wipe[tiab] OR wiping[tiab] OR flush[tiab] OR brush*[tiab])) AND ("Bacterial Load"[Mesh] OR "Colony Count, Microbial"[Mesh] OR "Colony-Forming Units Assay"[Mesh] OR "Microbiological Techniques"[Mesh] OR Colony-Forming Unit*[tiab] OR CFU[tiab] OR CFUs[tiab] OR Load[tiab] OR Contamination[tiab])

**The Cochrane Library: 14**

1. MeSH descriptor: [Laryngoscopes] explode all trees
2. MeSH descriptor: [Laryngoscopy] explode all trees
3. #1 OR #2
4. MeSH descriptor: [Endoscopes] explode all trees
5. MeSH descriptor: [Endoscopy] explode all trees
6. (Endoscop*):ti,ab,kw (Word variations have been searched)
7. #4 OR #5 OR #6
8. MeSH descriptor: [Otolaryngology] explode all trees
9. ((Ear-Nose-Throat OR ENT OR Otolaryngo* OR Otorhinolaryngo* OR pharynx OR nasal OR laryngoscop* OR pharyngolaryngoscop* OR pharyngoscop* OR Otorhinolaryngoscop*)):ti,ab,kw (Word variations have been searched)
10. #8 OR #9
11. #7 AND #10
12. MeSH descriptor: [Culture Techniques] explode all trees
13. ((Cultur* OR cultivation OR Sample-tak* OR Sample-collect* OR samples OR Sampling-method* OR wipes OR wipe OR wiping OR flush OR brush*)):ti,ab,kw (Word variations have been searched)
14. #12 OR #13
15. MeSH descriptor: [Bacterial Load] explode all trees
16. MeSH descriptor: [Colony Count, Microbial] explode all trees
17. MeSH descriptor: [Colony-Forming Units Assay] explode all trees
18. MeSH descriptor: [Microbiological Techniques] explode all trees
19. ((Colony-Forming Unit* OR CFU OR CFUs OR Load OR Contamination)):ti,ab,kw (Word variations have been searched)
20. #15 OR #16 OR #17 OR #18 OR #19
21. #11 AND #14 AND #20

**CINAHL: 29**

( ( (MH "Laryngoscopy") OR ( TI Laryngoscope OR AB Laryngoscope ) ) OR ( ( ( (MH "Endoscopes+") OR (MH "ENT Endoscopes") ) OR (MH "Endoscopy+") OR ( TI Endoscop* OR AB Endoscop* ) ) AND ( (MH "Diagnosis, Otorhinolaryngologic+") OR ( TI ( ear-nose-throat OR ENT OR otolaryngo* OR otorhinolaryngo* OR larynx OR pharynx OR Nasal OR laryngoscop* OR pharyngolaryngoscop* OR otorhinolaryngoscop* ) OR AB ( ear-nose-throat OR ENT OR otolaryngo* OR otorhinolaryngo* OR larynx OR pharynx OR Nasal OR laryngoscop* OR pharyngolaryngoscop* OR otorhinolaryngoscop* ) ) ) ) ) AND ( (MH "Culture Techniques+") OR (MH "Microbiological Techniques+") OR ( TI ( cultur* OR cultivation OR sample-tak* OR sample-collect* OR sample* OR sampling-method* OR wipe* OR wiping OR flush OR brush* ) OR AB ( cultur* OR cultivation OR sample-tak* OR sample-collect* OR sample* OR sampling-method* OR wipe* OR wiping OR flush OR brush* ) ) ) AND ( ( (MH "Colony Count, Microbial") OR (MH "Bacterial Contamination") ) OR (MH "Colony-Forming Units Assay") OR ( TI ( Bacterial load OR microbiological techniques OR colony-forming unit* OR CFU OR CFUs OR load OR contamination ) OR AB ( Bacterial load OR microbiological techniques OR colony-forming unit* OR CFU OR CFUs OR load OR contamination ) ) )

**Embase: 179**

(exp direct laryngoscopy/ or exp laryngoscopy/ or exp flexible fiberoptic laryngoscopy/ or exp flexible laryngoscopy/ or (laryngoscope/ or otolaryngology endoscope/ or fiberoptic laryngoscope/ or flexible laryngoscope/ or rigid laryngoscope/) or ((endoscope/ or exp endoscopy/ or endoscop*.ti,ab,kf.) and (otorhinolaryngology/ or (Ear-Nose-Throat or ENT or Otolaryngo* or Otorhinolaryngo* or pharynx or larynx or nasal or laryngoscop* or pharyngolaryngoscop* or pharyngoscop* or otorhinolaryngoscop*).ti,ab,kf.))) and (cell culture technique/ or cell culture/ or (microbiology/ or bacteriology/) or (Cultur* or cultivation or Sample-tak* or sample-collect* or samples or sampling-method* or wipes or wipe or wiping or flush or Brush*).ti,ab,kf.) and (bacterial load/ or exp bacterial count/ or cfu counting/ or microbiological examination/ or (Colony-Forming Unit or CFU or CFUs or load or Contamination).ti,ab,kf.)
