# Supplementary material for: Sampling methods for flexible endoscopes without a working channel: a scoping review
Source: Infect Control Hosp Epidemiol. 2025 Apr 21;46(6):635–40. doi: 10.1017/ice.2025.56 (PMC12169950; doi:10.1017/ice.2025.56)
Supplement: Halmans et al. supplementary material 3 — Halmans et al. supplementary material [file S0899823X2500056Xsup003.docx]

Table 2: Study characteristics

| Study | Country | Objective(s) | Endoscope type | Moment of sample taking | Sampling technique(s) | Study design | Key findings or opinions related to sampling the tip or shaft of flexible endoscope without a working channel | Bacteria found on tip or shaft | Type of culture plate and incubation time/Processing of samples |
| --- | --- | --- | --- | --- | --- | --- | --- | --- | --- |
| Abramson et al, 1993 | United States | (1) To determine the residual microbial load on rigid and flexible endoscopes after clinical use and after in vitro experimental inoculations. (2) To investigate the efficacy of Cidex-plus for disinfection. | 90-degree rigid laryngoscope, pediatric flexible laryngoscope or adult flexible laryngoscope | (1) After clinical examination.  (2) After laboratory contamination. (3) After disinfection. | Immersing the tip 10 times into a tube containing 5ml neutralizing buffer. | Controlled clinical trial | The immersion sampling technique was able to detect microorganisms after clinical use (1) and after laboratory contamination (2). After disinfection (3), only one flexible laryngoscope yielded microorganisms. | (1) Viridans streptococci, S. aureus, S. epidermidis, S. capitis, S. hominis, S. warneri, S. xylosis, Corynebacterium spp., C. albicans, Neisseria sicca, Moraxella catarrhalis, C. diversus, Proteus mirabilis, Kingella spp., Pseudomonas paucimobilis  (2) S aureus, E. Coli, P. aeruginosa, C Albicans, S sanguis  (3) S. mitis, Neisseria sicca | Blood agar plate, chocolate agar, Mitis-Salivarius agar and Selective Enterococcus agar at 35°C for 24-48 h. |
| Alvarado et al, 2009 | United States | To determine whether the use of a novel sheath can provide reliable protection against bacterial contamination and obviate the need for routine HLD. | Flexible nasopharyngoscopes | (1) Prior to application of the sheath and endoscopic examination. (2) Immediately after endoscopic examination after removal of the sheath. (3) After disinfection and air-drying. | Wiping the surface of the head or shaft with a sterile saline pledget. | Cross-sectional | The wiping sampling technique was able to detect bacteria after sampling time 1 and 2, but no bacteria were found after sampling time 3. | Detected at the shaft: (1) CoNS, Corynebacterium spp, Bacillus spp, (2) CoNS (3) No bacteria found | Plated on 5% sheep blood agar and incubated for 72 h at 37 °C. |
| Bhattacharyya et al, 2004 | United States | Determine whether HLD renders fiberoptic laryngoscopes free of nonviral infectious microorganisms. | Flexible fiberoptic laryngoscopes without biopsy channels | (1) At the start of the clinic, at noon, and at the end of the day  (2) Control cultures were taken after placing the laryngoscope into the oral cavity in direct contact with the mucous membranes. | Dipping the fiberoptic laryngoscope into sterile culture medium. | Cross-sectional | (1) The dipping method was able to detect Rhizopus spp., after clinical use and cleaning.  (2) The dipping method was able to detect microorganisms after direct contact with mucous membranes. | (1) Rhizopus spp  (2) oral flora and β-hemolytic streptococcal species | Not specified. |
| Chang et al, 2012 | United States | Determine the efficacy of various cleaning and disinfective methods in reducing bacterial and fungal load | FFL | (1) After in vitro contamination. (2) After disinfection with either 30-second antimicrobial soap scrub, 30-second scrub with 70% isopropyl alcohol, 30-second soap and water scrub followed by 30-s 70% isopropyl alcohol scrub, 30-s scrub with germicidal cloth, immersion in ortho-phthalaldehyde scrub (5, 10, 15 or 20 minutes) or isolated 30-second tap water rinse | Immersion in sterile saline. | In vitro | The immersion sampling technique was able to yield S. aureus and C. albicans after in vitro contamination. After disinfection, all but 2 out of 5 plates after an isolated 30-second tap rinse, showed no contamination. | (1) Staphylococcus aureus, Candida albicans  (2) Staphylococcus aureus, Candida albicans | Agar plate for 24-48 h. Temperature not specified. |
| Cottarelli et al, 2020 | Italy | Evaluate microbiological contamination of endoscopes after HLD, and the involvement of reprocessing procedures adopted in endoscopy units of an Italian teaching-hospital. | Bronchoscopes, gastroscopes, colonoscopes and faryngo-laryngoscopes | (1) Immediately after reprocessing (<1 hour). (2) after storage < 72 hours from reprocessing. (3) In storage for >72 hours | Samples of the outer surface were collected using sterile swabs moistened with sterile saline. | Cross-sectional, survey | In 4 out of 40 post-reprocessed broncho-laryngoscopes, indicator micro-organisms could be detected using the swab sampling technique. Not clear whether this contamination was seen at sampling point 1,2 or 3. | Pseudomonas aeruginosa, other gram-negative nonfermentant, S. aureus | 5ml of the sample was inoculated in Tryptic Soy Broth and incubated at 36 °C for 48 h and in case of bacterial growth streaked out on selective agar plates (Violet Red Bile Glucose agar for Enterobacteriaceae, Brilliance Salmonella Agar Base with  supplement for Salmonella spp, Pseudomonas  Cetrimide agar for P. aeruginosa, Mannitol Salt agar for S. aureus)  and incubated fat 37 °C for 48 h. For the detection of Legionella, the samples were plated on Buffered Charcoal Yeast Extract Agar supplemented with L-cysteine and MWY selective supplement and incubated at 36±1°C for 10 days. For the detection of mycobacteria the samples were inoculated into mycobacteria growth indicator tube liquid medium vials and Löwestain-Jensen slants and incubated at 36±1°C with an observation time of six weeks. |
| Ditommaso et al, 2019 | Italy | Evaluate the efficacy of HLD of FFNs with wipes impregnated with a chlorine dioxide solution against a conventional automated washer disinfector. | FFNs | (1) After clinical use and pre-disinfection. (2) After HLD | (1) Shaking the tip of the insertion shaft in a sterile collection tube with preservation medium. (2) Wiping the whole insertion tube with a sterile TNT wipe, pre-moistened with 0.5ml sterile water. | Controlled clinical trial | Both sampling techniques were able to yield bacteria pre- and post-disinfection | (1) S. epidermidis, S. aureus, CoNS, S. viridans, S. pneumoniae, Corynebacterium spp., Corynebacterium striatum, other gram-positive bacteria, Pseudomonas aeruginosa, S. maltophilia, Neisseria, H. influenzae, H. parainfluenzae, Enterobacteriaceae, Non-fermenting Gram-negative bacilli, other gram-negative bacteria, C. albicans  (2) S. viridans, S. epidermidis, Rothia mucilaginosa, S. hominis, Micrococcus luteus, Kocuria spp., H. influenza, S. pneumoniae, P. aeruginosa | Aerobic cultures: specific culture media for detection of main microorganisms of nasopharyngeal flora at 37 °C for 24-48 hours  Anaerobic cultures: incubated in jars fille with mixed gas at 37 °C for 7 days with examination every 48 h |
| Elackattu et al, 2010 | United States | Assess the efficacy of using a sterile sheath to prevent cross-contamination | FNPL | (1) Prior to use (2) After HLD disinfection.  OR  (1) Prior to use. (3) After clinical use and removal of disposable sheath. (4) After cleaning with alcohol wipe. | Swiping the handle and lower third of the insertion shaft with sterile cotton swabs and placing them in 3mL sterile water in a collection tube. | Controlled clinical trial | The swiping sampling technique was able to yield bacteria before clinical use (1). After HLD, the shaft showed no contamination (2). Both after sheath removal (3) and after cleaning with alcohol wipe, microorganisms were detected (4). | (1) CoNS, Bacillus, Streptococcus not group D, diphtheroid. (2) No bacterial detection. (3) CoNS, fungus. (4) Streptococcus not group D | Blood agar plates and incubated for 72 h, temperature not specified. |
| Hitchcock et al, 2016 | New Zealand | Compare the microbiological efficacy, turnaround time, costs, convenience, and patient and user tolerance of TTW, PeraSafe solution and Cidex OPA solution for HLD of flexible nasendoscopes. | Flexible nasendoscopes | (1) Prior to clinical use. (2) Immediately after HLD. | Swabbing the tip and handle. | Controlled clinical trial | (1) No information is given about the samples taken before clinical use. (2) After HLD, the swabbing method was able to detect microorganisms. | (2) CoNS | Not specified. |
| Liming et al, 2014 | United States | Evaluate and compare the efficacy of varying techniques of HLD. | FFL | (1) Positive control, no decontamination after clinical use  (2) After disinfection with either 30-second tap water wash, 30-second antimicrobial soap scrub, 30-second 70% isopropanol scrub, 30-second 70% isopropanol plus antimicrobial soap scrub, 30-second germicidal cloth scrub, 12-minute Cidex OPA soak, 15-minute Cidex OPA soak or 20-minute CidexOPA soak. | Submerging the distal end into 20 mL of tryptic soy broth. | Controlled clinical trial | Submerging into tryptic soy broth was able to detect microorganisms after clinical use, after disinfection using 30-second tap water wash and after 30-second antimicrobial soap scrub. No contamination was found following the other disinfection techniques. | Not specified. | Chocolate II agar, incubated at 35°C in 8% CO2 for 24 hours or Sabouraud dextrose plates, incubated at 35°C for 48 h. |
| Okano et al, 2022 | Japan | Assess EAW disinfection of FEs and its in vitro inactivation of coronavirus and bacteria. | Channeled or non-channeled otorhinolaryngological endoscopes | (1) After clinical use. (2) Immediately after disinfection. | FEs without a working channel: swabbing the flexible tube, tip and control handle with a wet bacteriological swab. | Cross-sectional | (1) The swabbing sampling technique was able to detect microorganisms after clinical use. (2) After disinfection, no bacteria were found on the outer surface (only within the suction channel). | (1) Staphylococcus, Cutibacterium, Corynebacterium, Streptococcus, Pseudomonas, Bacillus, Rothia, Serratia, Klebsiella, Citrobacter, Proteus, other | Not specified. |
| Phua et al, 2012 | United Kingdom | Compare the efficacy and cost-effectiveness of chlorine dioxide wipes versus automated washer | Flexible nasendoscopes | (1) Before in vitro contamination (2) After decontamination | Swabbing the tip. | In vitro | The swabbing sampling technique was able to show bacterial growth before in vitro contamination (1) and after decontamination (2). | (1) S. epidermidis, Bacillus, diphtheroid, α haemolytic streptococci, coliforms  (2) S. epidermidis, Bacillus, diphtheroid, α haemolytic streptococci, coliforms | Not specified. |
| Tzanidakis et al, 2012 | United Kingdom | Evaluate the efficacy of the Tristel wipes in decontaminating and identify significant contamination between cleaning and use. | Flexible nasendoscopes | (1) After disinfection. (2) Between the cleaning process and clinical use. | Swabbing the tip and handle. | Cross-sectional | The swabbing method was not able to yield microorganisms of the tip after cleaning with Tristel wipes or before using on the patient. | No bacteria detected from the tip (Only bacteria collected from the handle of the endoscope) | Not specified. |

Abbreviations: HLD = High level disinfection, FFNs= flexible fiberoptic nasofibroscopes, CoNS= Coagulase-negative staphylococci, EAW= Electrolyze Acid Water, FEs= flexible endoscopes, TTW= Tristel Trio Wipes, FNPL= Flexible nasopharyngolaryngoscopes, FFL= Flexible fiberoptic laryngoscopes
